# Association of reduced REM sleep with mortality in adults with coronary artery disease and obstructive sleep apnea in the RICCADSA cohort

**DOI:** 10.1007/s11325-026-03614-1

**Published:** 2026-02-20

**Authors:** Baran Balcan, Yeliz Celik, Erik Thunström, Helena Glantz, Patrick J. Strollo, Susan Redline, Yüksel Peker

**Affiliations:** 1 Department of Pulmonary Medicine, Koc University School of Medicine, Istanbul, Türkiye; 2 Koc University Research Center for Translational Medicine (KUTTAM), Koç University, Istanbul, Türkiye; 3 Department of Molecular and Clinical Medicine, Institute of Medicine, Sahlgrenska Academy, University of Gothenburg, Gothenburg, Sweden; 4 Region Västra Götaland, Department of Medicine, Geriatrics and Emergency Medicine, Sahlgrenska University Hospital/Östra, Gothenburg, Sweden; 5 Department of Internal Medicine, Skaraborg Hospital, Lidköping, Sweden; 6 University of Pittsburgh School of Medicine, Pittsburgh, PA USA; 7 Division of Sleep and Circadian Disorders, Brigham and Women’s Hospital, Harvard Medical School, MA Boston, USA; 8 Department of Clinical Sciences, Respiratory Medicine and Allergology, Faculty of Medicine, Lund University, Lund, Sweden

**Keywords:** Obstructive sleep apnea, Coronary artery disease, Mortality, REM sleep

## Abstract

**Purpose:**

Reduced rapid-eye movement (REM) sleep has been linked to increased mortality in the general population. We investigated whether diminished REM sleep is associated with higher mortality in adults with coronary artery disease (CAD) and obstructive sleep apnea (OSA).

**Methods:**

This secondary analysis of the RICCADSA trial included 356 revascularized CAD patients with OSA (apnea–hypopnea index [AHI] ≥ 15 events/h) and total sleep time (TST) ≥ 240 min on baseline polysomnography. Reduced REM sleep was defined as the lowest quartile of REM percentage. Cox proportional hazards models assessed the association between reduced REM sleep and mortality over a median 4.7-year follow-up.

**Results:**

The lowest REM quartile corresponded to 8.7% of TST. Participants with reduced REM sleep (*n* = 86) were older (66.0 ± 8.1 vs. 63.0 ± 8.0 years; *p* = 0.035), had higher BMI (29.8 ± 4.6 vs. 28.7 ± 3.8 kg/m²; *p* = 0.010), shorter TST (369 ± 77 vs. 497 ± 69 min; *p* < 0.001), less slow-wave sleep (5.2 ± 7.0% vs. 8.1 ± 10.0%; *p* = 0.007), and higher AHI (54.4 ± 26.3 vs. 35.6 ± 20.1 events/h; *p* < 0.001) than those with REM ≥ 8.7% (*n* = 270). Mortality was 12.8% in the reduced REM group versus 4.4% in the higher REM group (*p* = 0.006). Reduced REM sleep independently predicted mortality (hazard ratio 2.39; 95% CI 1.03–5.56; *p* = 0.043) after adjustment for age, sex, BMI, and CPAP allocation. Further adjustment for TST, slow-wave sleep, baseline AHI, coronary bypass surgery, atrial fibrillation, and REM–AHI interaction did not alter the association.

**Conclusions:**

Reduced REM sleep independently predicted higher all-cause mortality in revascularized CAD patients with OSA. Identifying diminished REM sleep may help identify particularly vulnerable patients.

**Supplementary Information:**

The online version contains supplementary material available at 10.1007/s11325-026-03614-1.

## Introduction

Obstructive sleep apnea (OSA), is characterized by repeated episodes of partial or complete upper airway obstruction during sleep, exacerbates systemic inflammation, oxidative stress, and sympathetic overactivity, all of which are critical drivers of cardiovascular morbidity [1]. Rapid-eye movement (REM) sleep, a distinct phase of the sleep cycle, is recognized as essential for maintaining various physiological and cognitive functions [2]. Moreover, it is characterized by increased brain activity, muscle atonia, and vivid dreaming, playing a pivotal role in emotional regulation, memory consolidation, and cardiovascular recovery during sleep. Disruptions in REM sleep duration have been linked to adverse health outcomes, including increased risk of hypertension, diabetes, and cardiovascular disease [3, 4]. Recent evidence suggests that reduced REM sleep is associated with an elevated risk of mortality in the general population [5].

CAD, a leading cause of death worldwide, involves the narrowing or blockage of coronary arteries, which reduces blood flow to the heart and increases the risk of myocardial infarction and heart failure [6]. Both CAD and OSA are independently associated with heightened mortality risks and increased cardiovascular burden [1, 7–10].

The association between reduced REM sleep and mortality risk in individuals with CAD and OSA is unknown. Understanding this relationship is essential for identifying at-risk populations and developing targeted interventions to improve sleep quality and cardiovascular outcomes. In the current study, we aimed to explore the specific role of reduced REM sleep regarding mortality among adults with CAD and OSA.

## Methods

### Study design, participants, group assignment, and follow-ups

This study represents a secondary analysis of data derived from the Randomized In-tervention with Continuous Positive Airway Pressure (CPAP) in Coronary Artery Disease and Sleep Apnea (RICCADSA) trial, a prospective, randomized controlled study conducted in Sweden between 2005 and 2013. The full trial design, methodology, and primary outcomes have been previously described [11].

**Fig. 1:**
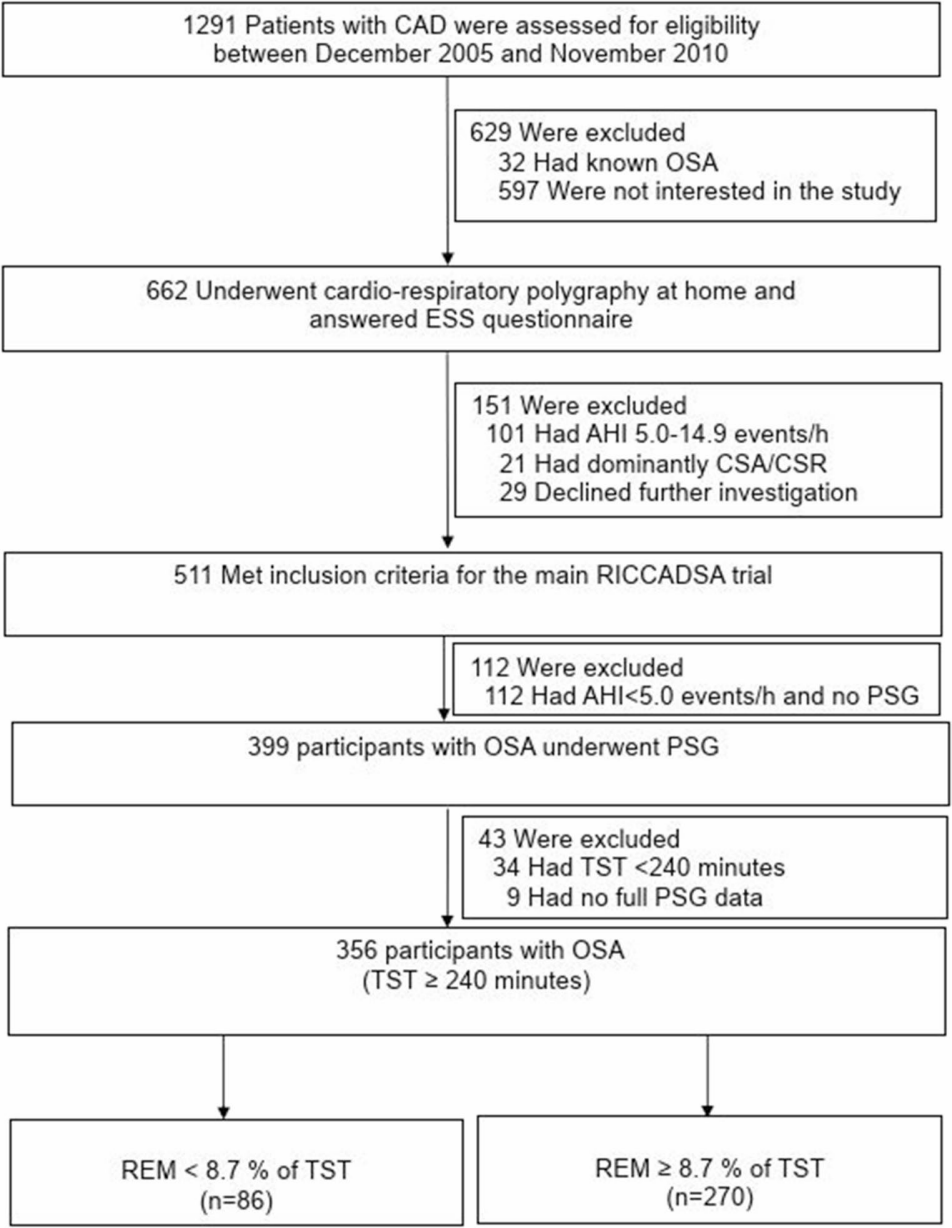
Flow of patients through the study. *Definition of abbreviations*: AHI, apnea-hypopnea index; CAD; coronary artery disease; CPAP, continuous positive airway pressure; CSA-CSR, central sleep apnea-Cheyne Stokes respiration; ESS, Epworth Sleepiness Scale; OSA, obstructive sleep apnea; PSG, Polysomnography; REM, Rapid Eye Movement; RICCADSA, Randomized Intervention with CPAP in Coronary Artery Disease and Sleep Apnea; TST, Total Sleep Time

In brief, revascularized patients were recruited from two hospitals with training and research facilities serving a population of approximately 250,000 living in the Skaraborg County of West Götaland, Sweden. Percutaneous coronary intervention (PCI) was performed either as an elective or acute/subacute procedure at the hospital in Skövde or at the Sahlgrenska University Hospital in Gothenburg, which is the regional hospital. Coronary artery bypass grafting (CABG) was performed in Gothenburg, and all patients were moved to the study hospitals in Skövde or Lidköping when clinically stable after revascularization. Patients were screened for OSA using home sleep apnea testing (HSAT) over one night and classified according to the apnea–hypopnea index (AHI) and Epworth Sleepiness Scale (ESS) scores. All HSAT recordings were validated by the same sleep physician (Y.P). Those with an ESS score ≥ 10 were classified as having OSA with excessive daytime sleepiness (EDS) and were offered auto-titrating CPAP therapy. Participants with an AHI of ≥ 15 events per hour and an ESS score < 10 were classified as having non-sleepy OSA and were randomized to receive either CPAP or no CPAP in the primary trial protocol. At baseline, all participants with OSA underwent polysomnography (PSG) in a hospital setting to provide a detailed assessment of their sleep architecture. Patients with a total sleep time (TST) of at least 240 min on the PSG (*n* = 356) were included in the current analysis (Fig. 1).

The percentage of REM sleep obtained from baseline PSG was used to categorize patients. The lowest quartile of REM sleep percentage (< 8.7%) defined the reduced REM group, while the remaining participants constituted the higher REM group. Follow-up continued for up to eight years, with a median of 4.7 years, during which all-cause mortality was recorded.

Follow-up data were obtained from national health registries and hospital medical records. Deaths were adjudicated by an independent Clinical Event Committee blinded to OSA status and CPAP allocation. Causes of death were verified against death certificates and classified using ICD-10 codes.

### Sleep recordings

#### Home sleep apnea testing (HSAT)

Initial OSA screening was conducted using the Embletta^®^ Portable Digital System, a validated type 3 HSAT device, consisting of the following: (1) nasal pressure detector using nasal cannulae/pressure transducer system, recording the square root of pressure as an index of flow; (2) thoraco-abdominal movement detection through two XactTrace™ inductive belts with respiratory inductance plethysmography (RIP) technology; (3) finger pulse oximeter detecting heart rate and oxyhemoglobin saturation (SpO2); and (4) body position and movement detection. The patient’s sleep time was estimated on the basis of self-reporting as well as the pattern of body movement during the sleep study. Apneas were defined as an almost complete (≥ 90%) cessation of airflow. Hypopneas were de-fined as a ≥ 50% reduction in thoracoabdominal movement and/or a ≥ 50% decrease in the nasal pressure amplitude for ≥ 10 s according to the Chicago criteria [12, 13]. In addition, the total number of significant oxyhemoglobin desaturations (decrease of ≥ 4% from the immediately preceding baseline) were scored, and the oxygen desaturation index (ODI) was calculated as the number of significant desaturations per hour of estimated sleep. Events with a ≥ 30% reduction in thoracoabdominal movement and/or a ≥ 30% de-crease in the nasal pressure amplitude for ≥ 10 s were also scored as hypopneas if there was a significant desaturation (≥ 4%). Patients with AHI ≥ 15 per hour of estimated sleep time, independent of symptom occurrence, were defined as having OSA.

#### Polysomnography (PSG)

All patients with CAD and a diagnosis of OSA based on the first HSAT screening investigation underwent unattended overnight PSG in hospital using a computerized recording system (Embla A10^®^, Embla, Broomfield, CO, USA) including three-channel electroencephalography (EEG [C4/A1, C3/A2, CZ/A1]), two-channel electrooculography (EOG), one-channel submental electromyography (EMG), bilateral tibial EMG and two-lead electrocardiogram (ECG) in addition to the cardiorespiratory channels as de-scribed for the HSAT system above. PSG recordings were scored by a certified sleep technologist from an accredited sleep lab (see acknowledgement), blinded to clinical data and baseline screening results from the previous HSAT recordings. Obstructive events on the PSGs were scored according to the same criteria applied for the HSATs.

### Follow-up visits and adherence to CPAP

OSA patients receiving CPAP treatment brought their device to the clinic at each scheduled follow-up visit (at 1 month, 3 months, 6 months, 1 year, and then yearly up to 6 years); monitoring settings and hours of CPAP use were obtained from the machines’ internal clocks and recorded. In total, 9 follow-up visits were scheduled with a minimum of 5 visits (up to 2 years). CPAP use was evaluated as average usage hours per night at each visit after study start multiplied by the percentage of CPAP nights (adjusted usage), using data downloaded from the CPAP devices. Patients who discontinued CPAP treatment were included as “zero” – usage in the analysis. In addition, pressure level, mask leak and residual AHI measures were noted. All necessary adjustments of the CPAP device and mask fittings were done according to clinical routines by the sleep medicine unit staff.

### Outcomes

The primary outcome of the current protocol was all-cause mortality from baseline to end of follow-up. Secondary outcomes included differences in sleep parameters, respiratory indices, and cardiovascular comorbidities between reduced and higher REM groups. Date and cause of death were verified through the Swedish National Population Registry to ensure completeness of follow-up. As previously described [11, 12], an In-dependent Clinical Event Committee reviewed all data obtained from the medical records and death certificates by the end of May 2013, blinded to group allocation. Overall mortality was based on the death certificates.

### Statistical analysis

All statistical analyses were conducted using SPSS version 28.0 (IBM Corp., Armonk, NY, USA). Continuous variables were tested for normality via the Kolmogorov–Smirnov test. Data are presented as means ± SD for normally distributed variables or medians (interquartile range) for skewed variables. Categorical variables are expressed as frequencies and percentages. Between-group comparisons were performed using independent-samples t-tests or Mann–Whitney U tests for continuous variables and Chi-square tests for categorical variables. Pearson’s correlation assessed linear rela-tionships between REM percentage and other continuous parameters (e.g., BMI, AHI, TST). Binary logistic regression models identified predictors of reduced REM sleep, first in univariate analyses and then in multivariate models adjusting for potential confounders (age, sex, BMI, and comorbidities). Cox proportional hazards regression was employed to assess the association between reduced REM sleep and all-cause mortality. - Reduced REM sleep was defined a priori as the lowest quartile of REM sleep percentage in this cohort. This quartile-based approach was chosen to avoid arbitrary cutoffs and to allow identification of individuals with markedly reduced REM sleep within the study population. Covariates entered into the models included age, sex, BMI, and CPAP allocation; a second fully adjusted model also included CABG history, atrial fibrillation, baseline AHI, and TST. Collinearity among covariates was assessed using variance inflation factors, with all VIF values < 2.0. As the primary aim of the current analysis was to examine baseline REM sleep architecture as a prognostic marker, CPAP adherence was not modeled as a time-dependent covariate in the mortality analyses. However, CPAP allocation was included in all multivariable models. The proportional hazards assumption was confirmed via Schoenfeld residuals. Kaplan–Meier survival curves were generated to visualize mortality differences, and significance was tested using the log-rank test. A *p* < 0.05 (two-sided) was considered statistically significant for all analyses.

## Results

Median follow-up to the mortality or the end of study was 57.0 months (43.0–72.0 months). The lowest quartile of REM sleep was 8.7% of the TST in the entire cohort. As shown in Table 1, participants with REM sleep < 8.7% were slightly older and had a higher body mass index (BMI) compared to those with REM sleep ≥ 8.7%. Obesity (BMI ≥ 30) was significantly more common in the group with less REM sleep. Moreover, a higher proportion of participants with REM sleep < 8.7% underwent coronary artery bypass grafting (CABG) at baseline and had a history of atrial fibrillation. Other characteristics, including the percentage of females, ESS scores, prevalence of diabetes mellitus (DM), hypertension, current smoking, and the use of antidepressive drugs, showed no significant differences between the two groups (Table 1).

**Table 1:** Comparison of clinical and demographic characteristics between participants with REM Sleep < 8.7% and REM Sleep ≥ 8.7%

	REM < 8.7% (*n* = 86)	REM ≥ 8.7% (*n* = 270)
Age*, years	67.3 (60.4–72.6)	63.9 (58.7–69.8)
BMI*, kg/m^2^	29.7 (26.7–32.2)	28.1 (25.9–30.4)
Obesity* (BMI ≥ 30), %	48.8	27.0
Female sex, %	11.6	14.4
CABG*, %	37.2	21.9
ESS score	8.5 (5.0–11.0)	8.0 (5.0–11.0)
EDS, %	46.5	38.9
Current smoker, %	11.6	17.0
Hypertension, %	65.1	60.0
Diabetes mellitus, %	27.9	22.6
History of Atrial Fibrillation*, %	26.7	14.4
Stroke, %	8.1	6.3
Pulmonary disease, %	10.5	5.9
Antidepressive drugs, %	5.7	5.4

Continuous data are presented as median and 25%-75% quartiles. Categorical data are presented as percentage. *Definitions of abbreviations*: *BMI* body mass index, *CABG* coronary artery by-pass grafting, *EDS* Excessive Daytime Sleepiness, *ESS* Epworth Sleepiness Scale, *REM* rapid eye movements

*p < 0.05

As illustrated in Table 2, participants with REM sleep < 8.7% showed significantly worse sleep parameters compared to those with REM sleep ≥ 8.7% having shorter TST, less amount of Slow-Wave-Sleep (SWS), longer sleep onset latency and lower sleep efficiency. AHI and ODI were significantly higher in the group with less REM sleep. Participants with reduced REM sleep exhibited higher REM-specific AHI and supine AHI compared to those with higher REM sleep (Table 2).

**Table 2:** Sleep parameters of the participants with REM Sleep < 8.7% and REM Sleep ≥ 8.7%

	REM < 8.7%	REM ≥ 8.7%	*p* value
Total sleep time, min	369.10 ± 77.34	406.63 ± 69.16	< 0.001
Sleep onset, min	20.25 ± 13.95	14.29 ± 9.4	0.001
Sleep efficiency, %	73.42 ± 10.91	81.19 ± 10.22	< 0.001
AHI, events per hour	54.34 ± 26.28	35.60 ± 20.09	< 0.001
ODI, events per hour	32.79 ± 20.55	19.34 ± 15.30	< 0.001
Slow-Wave Sleep, min	19.24 ± 8.25	31.25 ± 23.75	< 0.001
Slow-Wave Sleep, %	5.18 ± 1.6	8.05 ± 5.85	< 0.001
REM sleep, min	22.64 ± 10.65	64.10 ± 23.51	< 0.001
REM sleep, %	6.04 ± 2.3	15.74 ± 5.07	< 0.001
Arousal index, events per hour	64.46 ± 22.81	45.22 ± 18.89	< 0.001
REM AHI, events per hour	52.07 ± 24.91	40.21 ± 24.73	< 0.001
Supine time, min	147.8 ± 121.3	155.5 ± 116.2	0.599
Supine AHI, events per hour	65.9 ± 24.1	52.1 ± 24.9	< 0.001
Lowest O2 saturation	80.03 ± 6.60	82.06 ± 7.23	0.002
Average O2 desaturation	6.16 ± 1.85	5.51 ± 1.56	0.023
O2 saturation below 90%, min	34.93 ± 12.50	18.36 ± 4.4	< 0.001
O2 saturation below 90%, %	9.64 ± 3.80	4.69 ± 1.10	< 0.001
Heart rate, beat per minute	60.53 ± 8.91	58.06 ± 8.51	0.031

Continuous variables were summarized as means with standard deviations. *Definitions of abbreviations*: *AHI* apnea-hypopnea index, *REM* rapid eye movements, *ODI* oxygen desaturation index

**Fig. 2:**
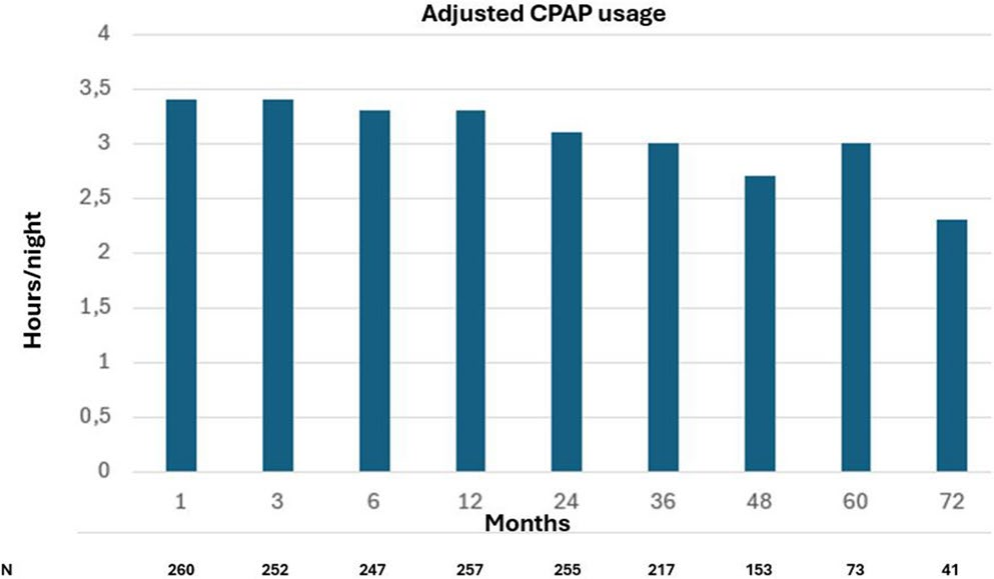
CPAP usage over time. *Definition of abbreviations*: CPAP, continuous positive airway pressure;

**Fig. 3:**
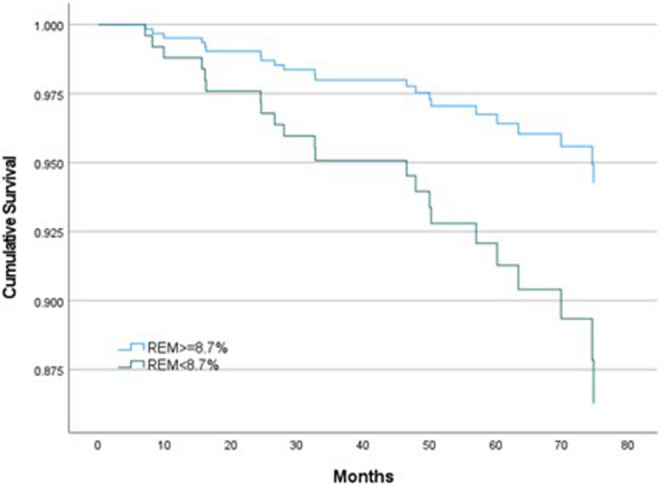
Kaplan-Meier curves for cumulative survival. *Definition of abbreviations*: REM, Rapid Eye Movement

Figure 2 illustrates the CPAP adherence data at each visit for participants allocated to CPAP. The average CPAP usage was 3.4 h per night at 1 month and 3.1 h at 2 years. As shown in Fig. 3, patients with reduced REM sleep exhibited significantly lower cumulative survival compared to those with REM sleep ≥ 8.7%, as illustrated in the Kaplan-Meier survival curve). Over the follow-up period, mortality was markedly higher in the group with less REM sleep (12.8%) than in those with higher REM sleep (4.4%; *p* = 0.006). In a multivariate Cox proportional hazard analysis adjusting for age, sex, BMI, and CPAP treatment allocation, reduced REM sleep was significantly associated with increased risk of mortality (HR 2.39; 95% CI, 1.03–5.56; *p* = 0.043) (Table 3). Age also emerged as a significant covariate (HR 1.07; 95% CI, 1.01–1.13; *p* = 0.028), whereas sex, BMI, and CPAP allocation did not demonstrate significant associations with mortality. The association between reduced REM sleep and mortality remained significant in the fully adjusted model adding CABG at baseline, history of atrial fibrillation, total TST, SWS, baseline AHI, and interaction between AHI and the lowest REM quartile (Table 3). All VIF values were below 2.0, indicating no clinically meaningful multicollinearity and supporting the stability of the hazard ratio estimates. To further explore whether the association followed a dose–response pattern, exploratory analyses across REM sleep quartiles were performed. These analyses did not demonstrate a significant graded relationship across quartiles, and the overall quartile effect was not statistically significant (data not shown).

**Table 3:** Multivariate regression analysis of the parameters related to mortality

Analysis 1	HR	95% CI	*p* value
Less REM Sleep	2.39	1.03–5.56	0.043
Age	1.07	1.01–1.13	0.028
Female sex	0.62	0.14–2.64	0.514
BMI	0.94	0.84–1.07	0.370
Allocation to CPAP	0.76	0.32–1.81	0.536
*Analysis 2*			
Less REM Sleep	3.28	1.09–9.93	0.035
Age	1.07	1.00–1.13	0.046
Female sex	0.74	0.17–3.36	0.699
BMI	0.96	0.85–1.08	0.506
Allocation to CPAP	1.00	0.39–2.55	0.998
CABG at baseline	0.94	0.36–2.44	0.894
History of AF	2.10	0.77–5.74	0.149
Total Sleep Time	1.01	1.00–1.01	0.033
Slow-Wave Sleep	0.99	0.97–1.03	0.559
AHI	1.01	0.99–1.02	0.632
Interaction Less REM Sleep & AHI	0.94	0.86–1.02	0.160

Definitions of abbreviations: *AF* Atrial Fibrillation, *AHI* Apnea Hypopnea Index, *BMI* Body Mass Index, *CABG* Coronary Artery Bypass Grafting, *CI* Confidence Interval, *CPAP* Continuous Positive Airway Pressure, *HR* Hazard Ratio, *REM* Rapid Eye Movements

## Discussion

The main finding of the current secondary analysis of the RICCADSA cohort demonstrated that the participants with REM sleep less than 8.7% at baseline had significantly higher mortality risk compared to the individuals with REM sleep at least 8.7%. Patients with reduced REM sleep was slightly older, had higher BMI, prior CABG, history of atrial fibrillation, shorter TST and higher AHI values, and the association of less REM sleep with mortality remained significant in the fully adjusted model.

To the best of our knowledge, this is the first study evaluating the relationship between less REM sleep and all-cause mortality in revascularized CAD patients with OSA. In a previous study by Leary et al. from two longitudinal studies, the Osteoporotic Fractures in Men (MrOs) and Wisconsin Sleep Cohorts, a significant association between reduced REM sleep and mortality was demonstrated [5]. Individuals in the MrOS cohort had a 13% higher mortality rate for every 5% reduction in REM sleep (percentage) after adjusting for multiple covariates. The findings were replicated in the Wisconsin Sleep Cohort despite younger age, inclusion of women, and longer follow-up [5]. This association persisted even after adjusting for total sleep time and other sleep stages, suggesting a specific role of REM sleep in survival outcomes. Further supporting this, Zhao et al. found that higher percentages of REM sleep and longer total REM sleep durations were associated with a reduced risk of incident heart failure [14]. Specifically, each 5% increase in REM sleep percentage corresponded to a 12% reduction in heart failure risk, highlighting the protective cardiovascular effects of adequate REM sleep [14].

We observed obesity more frequently in Less REM group and the median value of BMI was higher among those participants. Obesity hypoventilation syndrome (OHS) was not systematically diagnosed in this cohort, and arterial blood gas measurements were not part of the study protocol. However, there were no systematic indications of chronic hypercapnic respiratory failure. While we cannot fully exclude undiagnosed OHS, the prevalence is expected to be low, and any residual confounding would likely bias results toward the null. Poor REM sleep may lead to alterations in appetite-regulating hormones like leptin and ghrelin, promote insulin resistance, and contribute to weight gain. This creates a bidirectional relationship, as obesity itself is known to disturb sleep continuity and reduce REM duration [15, 16]. Disruption in REM duration may be linked to increased sympathetic drive, reduced vagal tone, and greater cardiovascular stress, potentially leading to cardiovascular arrhythmias, specifically supraventricular arrhythmias, as we observed a higher incidence of AF in the group with less REM sleep. Prior research supports these findings; for instance, reduced REM sleep has been independently associated with an elevated risk of AF, even when controlling for OSA and other confounders [17, 18]. Moreover, the elevated prevalence of prior CABG procedures among participants with less REM sleep suggests a relationship between disturbed sleep and advanced CAD. Evidence indicates that chronic sleep impairment accelerates atherosclerosis progression and increases the likelihood of coronary events [19]. These effects are mediated through systemic inflammation, endothelial dysfunction, and metabolic abnormalities [20].

Participants with REM sleep proportion of 8.7% or more exhibited significantly better sleep parameters compared to those with REM sleep less than 8.7%. Specifically, they had longer total sleep time, shorter sleep onset latency, higher sleep efficiency, lower AHI and ODI, fewer arousals, more delta sleep, improved oxygen saturation metrics, and slightly lower average heart rate. These findings align with existing literature on sleep architecture and its impact on overall sleep quality. For instance, higher sleep efficiency and longer TST are indicative of better sleep quality and are associated with improved cognitive and physiological functions [21]. Sleep deprivation has been shown to impair cognitive performance, highlighting the importance of adequate sleep duration and efficiency [21]. Lower AHI and ODI values suggest fewer breathing disturbances during sleep, which is crucial for maintaining optimal oxygen levels and reducing cardiovascular strain. Studies have demonstrated that nocturnal hypoxemia, as measured by desaturation patterns, is associated with increased cardiovascular mortality, underscoring the significance of these indices in assessing sleep-related breathing disorders [22]. Increased delta sleep, or slow-wave sleep, is essential for physical restoration and memory consolidation. Research indicates that slow-wave sleep plays a critical role in the reinforcement of declarative memory, involving coordinated activity between the neocortex and hippocampus [23]. Although participants with reduced REM sleep demonstrated higher REM-specific and supine AHI, the absence of a significant interaction between REM sleep duration and AHI suggests that the observed mortality risk was not solely driven by REM-predominant or positional OSA. Instead, reduced REM sleep itself appears to convey independent prognostic information.

To explore whether the association followed a dose–response pattern, we conducted exploratory analyses across REM sleep quartiles. These analyses did not demonstrate a significant graded relationship across quartiles, and the overall quartile effect was not statistically significant. Instead, the increased mortality risk appeared to be primarily driven by the lowest REM sleep quartile.

In the RICCADSA cohort, nocturnal hypoxemia quantified as hypoxic burden has recently been shown to be a strong and independent predictor of cardiovascular outcomes [24] While hypoxic burden reflects the cumulative depth and duration of oxygen desaturations, REM sleep represents a distinct neurophysiological state characterized by marked autonomic instability. The persistence of the association between reduced REM sleep and mortality after adjustment for OSA severity suggests that REM sleep duration and hypoxic burden may capture complementary, rather than redundant, dimensions of sleep-related cardiovascular risk.

It has also been argued that REM sleep in an individual patient could serve as a biomarker for general health [25]. Since an association does not necessarily imply causality, the relationship between reduced REM sleep and mortality may also reflect advanced CAD and more severe OSA as highlighted in the current report.

Despite the strengths of this study, several limitations should be acknowledged. First, as a secondary analysis of the RICCADSA trial, the study was not originally designed to investigate the specific role of REM sleep duration, which may limit causal inference. Second, although detailed PSG was conducted at baseline, sleep architecture was assessed using a single-night recording. This approach does not capture night-to-night variability in REM sleep, potentially affecting the accuracy of REM-related metrics. Third, while multivariate models adjusted for several important confounders—including age, BMI, and CPAP allocation—residual confounding cannot be ruled out, especially from unmeasured variables such as medication use, physical activity, or dietary habits. Of note, only a small proportion of the entire cohort were on antidepressive medication, thus, the probability of REM suppression effect of drugs as a confounding factor was low. Fourth, participants were selected based on a diagnosis of both CAD and moderate-to-severe OSA, which may limit the generalizability of findings to broader populations, including those with milder disease or without cardiovascular comorbidities. Future studies should aim to validate these findings using longitudinal designs with repeated sleep assessments and objective measures of treatment adherence.

## Conclusions

In conclusion, the findings highlight REM sleep as a critical component of both metabolic and cardiovascular health. Reduced REM sleep is associated with disrupted hormonal regulation, increased obesity risk, impaired sleep quality, and elevated mortality. Identifying diminished REM sleep may help recognize particularly vulnerable patients. Conversely, individuals with longer REM sleep duration demonstrate better overall sleep architecture, improved cardiopulmonary parameters, and significantly greater survival. These results emphasize the potential clinical value of identifying and targeting factors depressing REM sleep. Future strategies to preserve or enhance REM sleep—whether behavioral, pharmacologic, or device-based—may offer a novel pathway for mitigating OSA-related complications and improving long-term cardiovascular outcomes.

## Supplementary Information

Below is the link to the electronic supplementary material.

Supplementary Material 1

## Data Availability

Data collected for the study, including de-identified individual participant data will be made available to others within 6 months after the publication of this article, as will additional related documents (study protocol, statistical analysis plan, and informed consent form), for academic purposes (e.g., meta-analyses), upon request to the corresponding author (yuksel.peker@lungall.gu.se), and with a signed data access agreement.
